# Clinical predictors of anticipatory emesis in patients treated with chemotherapy at a tertiary care cancer hospital

**DOI:** 10.12669/pjms.322.9493

**Published:** 2016

**Authors:** Fawad Qureshi, Azhar Shafi, Sheeraz Ali, Neelam Siddiqui

**Affiliations:** 1 Dr. Fawad Qureshi, MBBS, FCPS (Medicine). Fellow Medical Oncology, Department Medical Oncology, Shaukat Khanum Memorial Cancer Hospital & Research Centre, Lahore, Pakistan. Department Medical Oncology, Shaukat Khanum Memorial Cancer Hospital & Research Centre, Lahore, Pakistan; 2 Dr. Azhar Shafi, MBBS, FCPS (Medicine). Fellow Medical Oncology, Department Medical Oncology, Shaukat Khanum Memorial Cancer Hospital & Research Centre, Lahore, Pakistan; 3 Dr. Sheeraz Ali, MBBS, FCPS (Medicine). Fellow Medical Oncology, Department Medical Oncology, Shaukat Khanum Memorial Cancer Hospital & Research Centre, Lahore, Pakistan; 4 Dr. Neelam Siddiqui, MBBS, FRCP. Department Medical Oncology, Shaukat Khanum Memorial Cancer Hospital & Research Centre, Lahore, Pakistan

**Keywords:** Anticipatory nausea and vomiting

## Abstract

**Objective:**

To determine the clinical predictors of anticipatory emesis in patients treated with chemotherapy at a tertiary care cancer hospital.

**Methods:**

This was a cross-sectional study conducted on 200 patients undergoing first line chemotherapy with minimum of two cycles at inpatient department and chemotherapy bay of Shaukat Khanum Memorial Cancer Hospital and Research Centre Pakistan. Anticipatory nausea and vomiting develops before administration of chemotherapy. Clinical signs and symptoms in patients with or without anticipatory emesis were compared using chi square test statistics.

**Results:**

The mean age of the study participants was 36.68 years (SD±12.23). The mean numbers of chemotherapy cycles administered were 3.23 (SD±1.2). Chemotherapy related nausea and vomiting was experienced by 188 (94%) patients and anticipatory nausea vomiting was reported in 90 (45%) of patients. Greater proportions of patients with anticipatory emesis were females. Fourteen (15.5%) p-value=0.031 patients with anticipatory emesis had history of anxiety and depression. Fifty nine (65.5%) p-value =< 0.0001 patients with anticipatory emesis had severe nausea after last chemotherapy cycle. Forty six (51.11%) p=<0.0001 patients had motion sickness.

**Conclusion:**

Female gender, history of motion sickness, anxiety and depression, severe nausea and vomiting experienced in pervious cycle of chemotherapy were clinical predictors of anticipatory nausea and vomiting.

## INTRODUCTION

Advent of new antiemetic regimens^1^ and wide spread availability of guidelines^2^-^5^ for chemotherapy induced nausea and vomiting is an integral component of oncology care. Ineffective control of nausea and vomiting can have serious medical and psychological repercussions. It can cause dehydration, wound dehiscence, severe anxiety, depression and loss of control.^6^ It can also result in treatment delays and can adversely affect compliance, health resource utilization and costs.^7^ Some authors have used different composites end points to asses chemotherapy induced nausea and vomiting and the use of rescue antiemetic as the primary end point.^8^

Nausea and vomiting occurring after the use of chemotherapeutic drugs has been a well-recognized fact for some time. Recently the focus has been on nausea and vomiting occurring before actually administering the chemotherapy.^9^ This is called anticipatory emesis which develops when the patient thinks about the treatment, nurse, doctor, the sight of hospital, chemotherapy bay or drugs. This can become a significant problem resulting in psychological barriers to treatment.^10^

Characteristics of anticipatory nausea and emesis suggest that it may be due to a learning model. Conditioned stimuli (like sights, sounds, or peculiar smell of the hospital) are present while the chemotherapy drugs (unconditioned stimulus) that produce the unconditioned response (nausea and emesis) are administered. After a few cycles of chemotherapy the conditioned response stimuli like sights, sounds and even thoughts of the hospital can be learned and they then can generate the conditioned response of nausea and vomiting. The frequency of such episodes increases with the number of chemotherapy cycles given and is related to both the frequency and severity of post treatment nausea and vomiting.^11^

Anticipatory nausea and vomiting is usually refractory to standard antiemetic therapy.^12^ On the other hand anti emetics have been found to paradoxically worsen symptoms, perhaps by providing conditioned stimulus themselves.^9^ However behavioral approaches such as systematic desensitization and psychotherapy may be helpful in some cases.^13^ Early identification of susceptible patients is very important as it may help to improve compliance and subsequently improve outcomes as well.

The aim of present study was to identify the clinical predictors for patients who are susceptible to anticipatory nausea and vomiting with accuracy and at an early stage so that any behavioral intervention may be helpful and make chemotherapy a well tolerated experience.

## METHODS

This was a cross-sectional study, conducted in chemotherapy bay and inpatient department of Shaukat Khanum Memorial Cancer Hospital and Research Centre Pakistan. Sample size was calculated by using WHO sample size calculator taking confidence level 95%, population proportion 31.5%. Absolute precision 5%, sample size (n) = 200. Patients of either genders, more than 18 years of age, undergoing first line chemotherapy for histopathology/cytology proven malignancy requiring chemotherapy with minimum two cycles were included in the study. Those patients who had medical conditions that can cause emesis like gastric outlet obstruction, gastritis, metastatic hepatic cancer, renal failure, hepatic dysfunction, metastatic brain disease, intestinal obstruction, were excluded. Anticipatory emesis was defined as nausea and vomiting occurring before a new cycle of chemotherapy in response to conditioned stimuli such as smell, sight and sound of the treatment room. Severity of nausea and vomiting was assessed by common terminology criteria for adverse events (CTCAE) version 4.0. Patients who had nausea and dizziness induced by motion, as in travel by car, bus, aircraft, were taken as positive for motion sickness. Anxiety and depression was assessed and confirmed by a psychiatrist. All patients who met inclusion criteria were enrolled in the study and informed consent was taken. Patients were assessed before chemotherapy regarding age, gender, marital status, education status, socioeconomic status, motion sickness, anticipatory emesis, anxiety and depression. All patients were asked during and after last chemotherapy regarding sensation of warmth, drug taste, generalized weakness, sweating, nausea vomiting. These events were recorded in a pre-defined proforma.

### Data analysis

The data was analyzed using statistical package for social sciences (SPSS) version 19. Categorical data (i.e. gender, marital status, education status, socioeconomic status, chemotherapy related nausea/vomiting, tumor site and clinical signs and symptoms were noted as frequency and percentage. Quantitative variables (i.e. age, weight, height, body mass index and number of cycles) were presented as mean ± standard deviation. Clinical signs and symptoms in patients with or without anticipatory emesis were compared using chi square test statistics. P-value less than 0.05 was considered significant.

## RESULTS

Two hundred study participants satisfying the inclusion and exclusion criteria were included in the study. The baseline characteristics of the study participants are shown in Table-I. Chemotherapy related nausea and vomiting was experienced by 188 (94%) of the participants and anticipatory emesis was present in 90 (45%) patients.

**Table-I:** Baseline characteristics of study participants.

Baseline Characteristics	Mean ± SD or N (Percentages)
Age (Years)	36.28 ± 12.23
*Gender*	
Male	90 (45%)
Female	110 (55%)
*Marital Status*	
Single	32 (16%)
Married	168 (84%)
*Education*	
Educated	123 (61.5%)
Illiterate	77 (38.5%)
*Socioeconomic Status*	
Low	141 (70.5%)
Middle	50 (25%)
High	9 (4.5%)
Body Mass index (Kg/m^2^)	23.82 ± 6.19
Number of Cycles	3.23 ± 1.2
Chemotherapy related Nausea/Vomiting	188 (94)

The majority of study participants enrolled in the study had breast cancer (29%), followed by hematological malignancy (28%) and genitourinary/gynecological malignancies (26%). The other less prevalent tumors identified were sarcomas (10%), GIT (6%) and lung (1%).

When clinical signs and symptoms in patients with and without anticipatory emesis were compared, only gender, history of anxiety and depression, motion sickness, severity of nausea after last treatment and severity of vomiting after last treatment were found significant. Patients with anticipatory emesis were females 58 (52.7%)/males 32 (35.5%). Fourteen (15.5%) patients with anticipatory emesis had history of anxiety and depression. Fifty nine (65.5%) patients with anticipatory emesis had severe nausea after last chemotherapy cycle. Thirty nine (43.33%) patients with anticipatory emesis had severe vomiting after last chemotherapy cycles. Forty six (51.11%) patient had history of motion sickness. Patients receiving second cycle of chemotherapy experienced more anticipatory emesis 43 (47.7%). The comparison of clinical signs and symptoms of patients with and without anticipatory emesis are shown in Table-II.

**Table-II:** In patients experiencing anticipatory emesis.

Clinical Symptoms	Anticipatory Emesis N (%) (90 patients)	No Anticipatory Emesis N (%) (110 patients)	P-value
*History of Anxiety/Depression*			
Yes	14 (15.5%)	06(5.45%)	0.016
No	76 (84.5%)	104 (94.54%)	
*Taste of Drug During Last Chemotherapy*			
Yes	66 (73.33%)	72 (65.45%)	0.148
No	24 (26.66%)	38 (34.54%)	
*Severity of Nausea after last R_X_*			
Moderate/Severe	59 (65.5%)	44(40%)	<0.0001
Mild or None	31(34.5%)	66(60%)	
*Severity of Vomiting after last R_X_*			
Moderate/Severe	39(43.33%)	30(27.27%)	<0.0001
Mild or None	51(56.66%)	80(72.72%)	
*Warmth sensation after last R_x_*			
Yes	8 (8.8%)	8 (7.84%)	0.795
No	82 (91.11%)	102 (92.72%)	
*History of Motion Sickness*			
Yes	46 (51.11%)	8 (7.27%)	<0.0001
No	44(48.88%)	102 (92.72%)	
*History of Psychiatric Illness*			
Yes	1 (1.11%)	1 (0.90%)	1.00
No	89 (98.88%)	109(99.09%)	
*Sweating after Last R_x_*			
Yes	6 (6.66%)	6 (5.45%)	0.771
No	84 (93.33%)	104(94.54%)	
*Generalized Weakness after last R_x_*			
Yes	54 (60%)	51 (46.36%)	0.037
No	36 (40%)	59 (53.63%)	

Chi square test were use.

## DISCUSSION

Despite various available guidelines^2^-^5^ chemotherapy induce nausea and vomiting continues to be one of it’s most common side effect.^1^-^5^ Recent studies have confirmed that with the use of guidelines based antiemetic regimens, control of chemotherapy induced vomiting is much better than nausea.^15^-^16^ In our study we evaluated patient characteristics and clinical features associated with anticipatory nausea and vomiting. Anticipatory nausea and vomiting is a multifaceted process. It has been speculated that anxiety may facilitate the development and potential expression of anticipatory side effects of chemotherapy.^13^ In our study we found female gender, history of anxiety, depression and motion sickness as major risk factors for development of anticipatory nausea and vomiting. Patients on second cycle of chemotherapy experienced more anticipatory nausea and vomiting then the first cycle. In our study education level, socioeconomic and marital status did not appear to be critical factors for development of anticipatory nausea and vomiting as in previous studies.^17^ Direct measures of anxiety and the potential of administered drugs to cause side effects could be used to test the potential involvement of these factors in the etiology of anticipatory side effects. The neural pathway between the vomiting center and the vestibular system has been implicated in the nausea, vomiting and motion sickness.^18^ An involvement of the vestibular system in the development of anticipatory side effects is suggested by the findings that a susceptibility to motion sickness had a higher specificity than any other single characteristic. Patients with motion sickness have more post treatment nausea and vomiting than those patients who are not suffering from motion sickness.^19^ The potential role of the vestibular system in anticipatory side effects suggests that antiemetics with a primary effect on the vestibular system (such as the antimotion drugs scopolamine or antihistamine class agents) might be effective in the control of anticipatory side effects for some patients. Patients at high risk for anticipatory side effects may benefit from intensified pharmacologic side effect management. The behavioral counter conditioning procedure of systematic desensitization has been shown to be effective in the management of anticipatory side effects once they have developed.^13^

Patients might benefit from changing clinical environments during treatments. For example, patients found to have a higher probability for developing anticipatory or conditioned nausea/vomiting may benefit from being treated in a different area of the clinic by different clinic personnel at each visit. It may also be reasonable to suggest to the patients that they use a different route each time they travel to the clinic to receive chemotherapy. Patients may further benefit by having their chemotherapy treatments on a more random schedule of times during the day or days during the week instead of the more usual practice of receiving treatments on the same day of the week at approximately the same time of day. Further studies are required on larger scale to identify risk factors and control of anticipatory nausea and vomiting.

## CONCLUSION

In our study female gender, motion sickness, anxiety/depression, severe nausea and vomiting experienced in pervious cycle of chemotherapy were strong clinical predictors of anticipatory nausea and vomiting. Early detection and behavioral intervention may be helpful, towards better tolerance of chemotherapy.
